# An epidemic context elicits more prosocial decision-making in an intergroup social dilemma

**DOI:** 10.1038/s41598-022-22187-z

**Published:** 2022-11-21

**Authors:** Magdalena Rychlowska, Job van der Schalk, Antony S. R. Manstead

**Affiliations:** 1 School of Psychology, Queen’s University Belfast, David Keir Bldg, 18-30 Malone Rd, Belfast, BT9 5BN UK; 2 School of Psychology, Cardiff University, Cardiff, UK

**Keywords:** Psychology, Human behaviour

## Abstract

Societal challenges such as the COVID-19 pandemic have the quality of a social dilemma, in that they compel people to choose between acting in their own interests or the interests of a larger collective. Empirical evidence shows that the choices people make in a social dilemma are influenced by how this decision is framed. In four studies, we examined how context of an epidemic influences resource allocation decisions in a nested social dilemma task, where participants share resources between themselves, their subgroup, and a larger collective. Participants consistently allocated more resources to the collective in the context of the Ebola epidemic than in the context of a neighborhood improvement project, and these choices were strongly associated with prescriptive social norms. Together, the findings provide an experimental demonstration that the context of a quickly spreading disease encourages people to act more prosocially.

## Introduction

COVID-19 has elicited examples of both selfish and prosocial behavior^1–5^. In response to this pandemic, people have had to make decisions that have a social dilemma quality, pitting their individual interests against those of the collective^6^. For example, in relation to social distancing, should we prioritize personal needs to socialize with loved ones or look after others’ interests by avoiding social contact? In the grocery store, should individuals stock up on products or consider the interests of vulnerable others by limiting purchases? Here we focus on how an epidemic, as a contextual factor in a social dilemma, influences prosocial behavior.

The contexts in which social dilemmas are encountered affect how people respond to them. For example, greater exposure to others’ suffering following a devastating cyclone has been shown to predict more generosity towards outgroups^7^. Relatedly, social cohesion typically increases following natural disasters or mass tragedies^8^, increasing the likelihood of unselfish behavior. One interpretation of such findings is that different situations activate different social norms. This may be particularly true for events posing an existential threat, such as a pandemic.

There are reasons to think that pandemics and epidemics activate ‘benevolence’ norms^9^. Historians have examined the ways in which people responded to such contexts through the ages. Cohn’s^10^ overview of this work led him to conclude that “across the wide sweep of recorded epidemics, blame and persecution were not the usual outcomes” (p. 533). Instead, he notes that “some of the most feared and deadly diseases (…) provoked waves of compassion and volunteerism” (p. 535). There have also been significant changes in social policy resulting from pandemics, for example, the Spanish flu epidemic of 1918 influenced attitudes to welfare and health provision, being partly responsible for the creation of the National Health Service in the United Kingdom^11^.

In considering why a pandemic should encourage prosocial behavior, it is instructive to examine findings on the impact of globalization on cooperation. Buchan and colleagues^12^ found that the degree to which an individual or a country participates in global economic, social, and cultural relations is associated with increased cooperation. Thus, the interconnectedness accompanying globalization encourages cooperation across traditional group boundaries. With a quickly spreading disease, the health of individuals is interdependent with that of anonymous others. This interconnectedness may induce a sense of shared fate and thereby promote societal cooperation^13^. Exposure to traumatic events that create a need for coordinated responses also increases a sense of shared fate, even if there have been previous divisions between social groups^14–16^.

**Figure 1:**
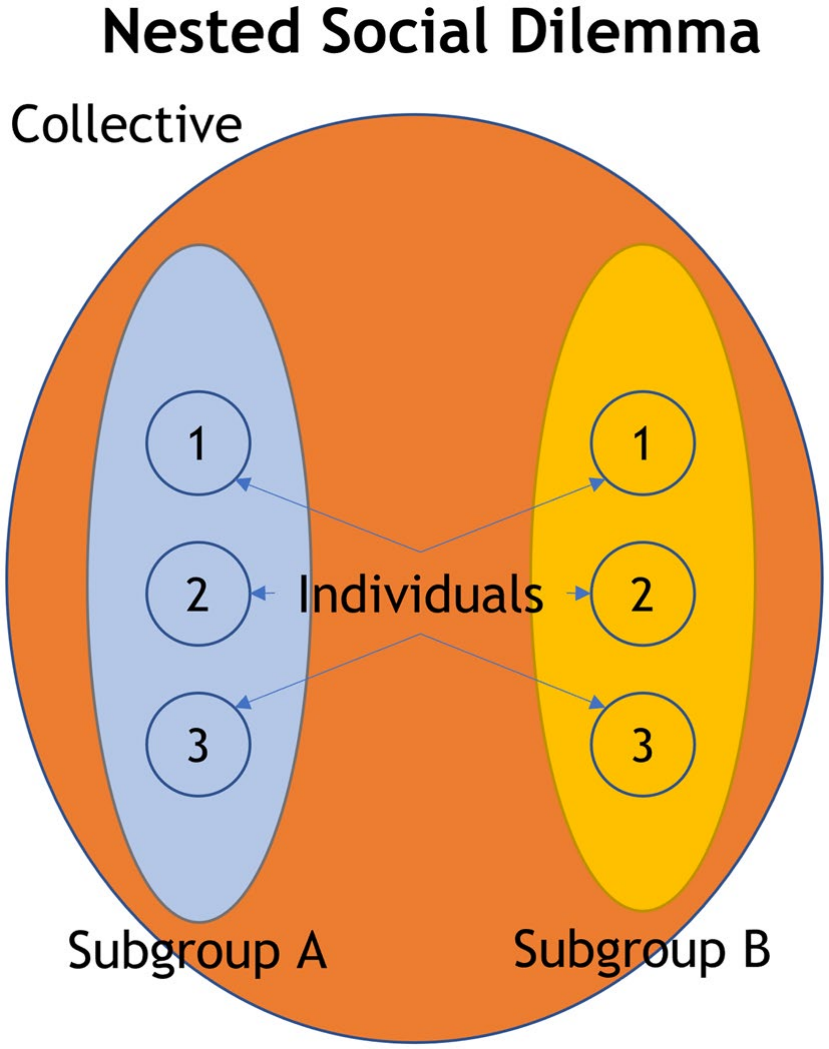
The structure of the Nested Social Dilemma (NSD) paradigm. Individuals (1, 2, 3) are members of one of two subgroups (Subgroup A or Subgroup B). The two subgroups are nested within a larger collective. Participants’ task is to divide an endowed resource between ‘accounts’ that would benefit themselves as individuals, their own subgroup, or the larger collective. The nature of the dilemma is that, although allocations to the subgroup and collective accounts are multiplied by a factor (2.5 and 4, respectively) before being divided between individual members, it is unclear how other participants will make their allocations.

To examine whether epidemics increase cooperative behavior relative to other contexts, we conducted a series of studies using the nested social dilemma (NSD) paradigm (see Fig. 1)^17^. In this decision-making task people divide resources between accounts reflecting different types of interest: themselves as individuals; a subgroup to which they belong; and a collective to which they and another subgroup belong. Contributions to subgroup and collective accounts are automatically multiplied, as an incentive to donate to these accounts, such that contributing to the subgroup account can be more profitable than keeping resources for oneself, and contributing to the collective account potentially offers even greater dividends. However, giving to the subgroup and collective accounts is risky because contributions to these accounts are distributed equally between all members and other players might contribute little or nothing while still profiting from others’ contributions. Giving to the individual account is the only way to be certain of your returns.

The NSD offers an elegant way of studying how people make decisions when the interests of individuals are pitted against those of subgroups and collectives. The task can readily be adapted to different settings^12^, which makes the NSD well suited to studying the influence of the context on decision-making.

### Overview of the present studies

In the original study where the NSD was first introduced, Wit and Kerr^17^ varied the level of categorization (individual, subgroup, or collective) that was salient to participants. To achieve this, the chance for participants to gain a cash reward was ostensibly determined in one of three ways: separately for each participant, separately for each subgroup, or for the entire collective. Making one of these levels salient was intended to increase participants’ concern for the individual, subgroup, or collective interests, respectively, and this was reflected in their participants’ allocations to the corresponding accounts. Our initial goal was to replicate these findings using a structurally identical NSD task in two contexts, one involving a neighborhood improvement project, and the other a campaign to protect public health during the 2013–2016 Ebola epidemic. In both contexts, participants were endowed with a monetary resource and divided it among the individual, the subgroup, and the collective accounts. In Studies 1 and 2 we manipulated category salience in a similar way to Wit and Kerr. In Study 3, we compared the neighborhood improvement and epidemic contexts directly and also examined the effects of information about outgroup behavior and emotion^18^. The rationale for these additional manipulations was to gain insight into the processes underlying different responses to epidemic and non-epidemic contexts, given that decisions made in social dilemmas are shaped by behavior of other people, including their emotional reactions (e.g.^18–21^). Because decision-making in social dilemmas is influenced by both expectations of how others will behave^18–22^ and by perceived social norms^9^, all studies measured expectations of how the ingroup and the outgroup would behave, and perceptions of how the ingroup *should* behave.

Studies were conducted between October 2014 and November 2016. In Study 1 we replicated Wit and Kerr’s findings using the neighborhood improvement context. In Study 2, we examined whether the same pattern of results would be observed in the context of the Ebola epidemic. At the time of the study (October–December 2014), Ebola was raging in West Africa, causing serious disruption. We sought to match the procedures used in Study 1, simply changing the context to the Ebola situation. Unexpectedly, we did not observe any effect of category salience in the epidemic context. Instead, in the epidemic context allocations to the collective account were greater than those to the individual or subgroup accounts. We replicated the findings of Study 2 in a separate experiment using a sample of British participants from the general population who were recruited via an online survey platform (Study S1, see Supplemental Materials). We then directly compared the neighborhood improvement context with the epidemic context to investigate whether the epidemic context increases participants’ allocations to the collective account in Study 3. We speculated that the context in which the NSD is presented might also affect expectations about outgroup allocations^18–22^. We therefore manipulated outgroup behavior, by providing information that a member of the ‘other’ subgroup had made a small allocation to the collective account, and felt either good or bad about doing so.

## Results

### Study 1

#### Allocations

Participants’ allocations to the individual account were influenced by the category salience manipulation, *F*(2, 131) = 19.33, *p* < 0.001, η_p_^2^ = 0.23, and were significantly higher in the individual condition (*M* = 263.11, *SD* = 117.43) than in the two-subgroups (*M* = 152.50, *SD* = 103.85) and the one-collective (*M* = 135.98, *SD* = 89.80) conditions, both *p*s < 0.001. The two-subgroups and one-collective condition did not differ significantly, *p* = 0.74. All pairwise comparisons used the Tukey HSD correction.

Allocations to the subgroup account were also affected by the experimental condition, *F*(2, 131) = 28.63, *p* < 0.001, η_p_^2^ = 0.30, with allocations in the two-subgroups condition (*M* = 233.33, *SD* = 133.52) significantly higher than allocations in the one-collective (*M* = 117.07, *SD* = 81.67) and the individual (*M* = 84.33, *SD* = 67.71) conditions, both *p*s < 0.001. The individual and one-collective conditions did not differ significantly, *p* = 0.29.

Allocations to the collective account were also affected by condition, *F*(2, 131) = 15.03, *p* < 0.001, η_p_^2^ = 0.19. Allocations in the one-collective condition (*M* = 246.95, *SD* = 130.41) were significantly higher than in the individual (*M* = 152.56, *SD* = 114.58) and two-subgroups (*M* = 114.17, *SD* = 103.99) conditions, *p* = 0.001 and *p* < 0.001, respectively. The individual and two-subgroups conditions did not differ significantly, *p* = 0.25. Expectations of ingroup and outgroup allocations and ingroup norms followed a similar pattern to the allocations to the different account types (see Supplemental Materials for details).

#### Correlations between allocations, expectations, and norms

Table 1 displays correlations between participants’ allocations to the collective account, and their expectations and prescriptive norms about allocations to this account. We focus on participants’ contributions to the collective account, on the grounds that this reflects a non-parochial form of cooperation^12^. Allocations were positively correlated with participants’ expectations of outgroup and ingroup allocations, and their perceptions of how much should be allocated to collective account. A multiple regression analysis showed that these three variables were significant predictors of allocations (see Supplemental Materials).

**Table 1:** Means (M), standard deviations (SD) and correlations between allocations to collective account, expectations of others’ allocations to this account, and prescriptive norms (Study 1).

	*M*	*SD*	1	2	3	4
1. Allocations	167.69	127.78	–			
2. Expectations—outgroup	120.78	101.88	0.62***	–		
3. Expectations—ingroup	126.42	108.86	0.69***	0.75***	–	
4. Norms—ingroup	206.34	149.02	0.69***	0.49***	0.55***	–

***p < 0.001.

### Study 2

#### Allocations

Allocations to the individual account, *F*(2, 123) = 0.45, *p* = 0.64, η_p_^2^ = 0.01, and the collective account, *F*(2, 123) = 1.85, *p* = 0.16, η_p_^2^ = 0.03, were not affected by the category salience manipulation. However, the effect of condition on allocations to the subgroup account was significant, *F*(2, 122) = 9.00, *p* < 0.001, η_p_^2^ = 0.13, with allocations in the two-subgroups condition (*M* = 146.34, *SD* = 108.77) being higher than allocations in the one-collective (*M* = 48.13, *SD* = 74.98) and (marginally) the individual (*M* = 97.39, *SD* = 121.23) conditions, *p* < 0.001 and *p* = 0.08, respectively. Allocations were also marginally higher in the individual than in the one-collective condition, *p* = 0.08.

Although the category salience manipulation had no consistent effect on allocations, allocations varied as a function of account type, regardless of condition, χ^2^_F_(2) = 47.33, *p* < 0.001. Allocations to the collective account were significantly higher (*M* = 275.99, *SD* = 162.55) than those to the individual (*M* = 127.10, *SD* = 131.73) and subgroup accounts (*M* = 97.68, *SD* = 110.70), *T* = 4933.00, *z* = − 5.39, *p* < 0.001 and *T* = 4429.00, *z* = − 6.57, *p* < 0.001, respectively. Allocations to the individual and subgroup accounts did not differ significantly, *T* = 1409.00, *z* = − 1.69, *p* = 0.09. Results for expectations of ingroup and outgroup allocations and ingroup norms can be found in Supplemental Materials.

#### Correlations between allocations, expectations, and norms

Table 2 shows correlations between participants’ allocations to the collective account and their expectations and norms about allocations to this account. Consistent with the findings of Study 1, these measures were positively correlated. A multiple regression analysis showed that expectations about ingroup allocations and ingroup norms were significant predictors of allocations (see Supplemental Materials for details).

**Table 2:** Means (M), standard deviations (SD) and correlations between allocations to collective account, expectations of others’ allocations to this account, and prescriptive norms (Study 2).

	*M*	*SD*	1	2	3	4
1. Allocations	275.99	162.55	–			
2. Expectations—outgroup	167.09	155.02	0.46***	–		
3. Expectations—ingroup	129.79	129.99	0.49***	0.42***	–	
4. Norms—ingroup	347.62	168.02	0.73***	0.43***	0.25*	–

***p < 0.001.

### Study 3

#### Allocations

Allocations to the collective account were analyzed using a 2 (context: neighborhood, Ebola) × 4 (information about outgroup contribution: no information; low allocation to collective account; low allocation and positive emotion; low allocation and negative emotion) between-subjects ANOVA. Allocations were higher in the Ebola context (*M* = 216.94, *SD* = 137.57) than in the neighborhood context (*M* = 179.21, *SD* = 121.70), *F*(1, 324) = 6.39, *p* = 0.01, η_p_^2^ = 0.02. The main effect of outgroup contribution failed to reach conventional levels of significance, *F*(3, 324) = 2.49, *p* = 0.06, η_p_^2^ = 0.02. The interaction between context and information about outgroup contribution was also not significant, *F*(3, 324) = 0.21, *p* = 0.89, η_p_^2^ < 0.01.

**Figure 2:**
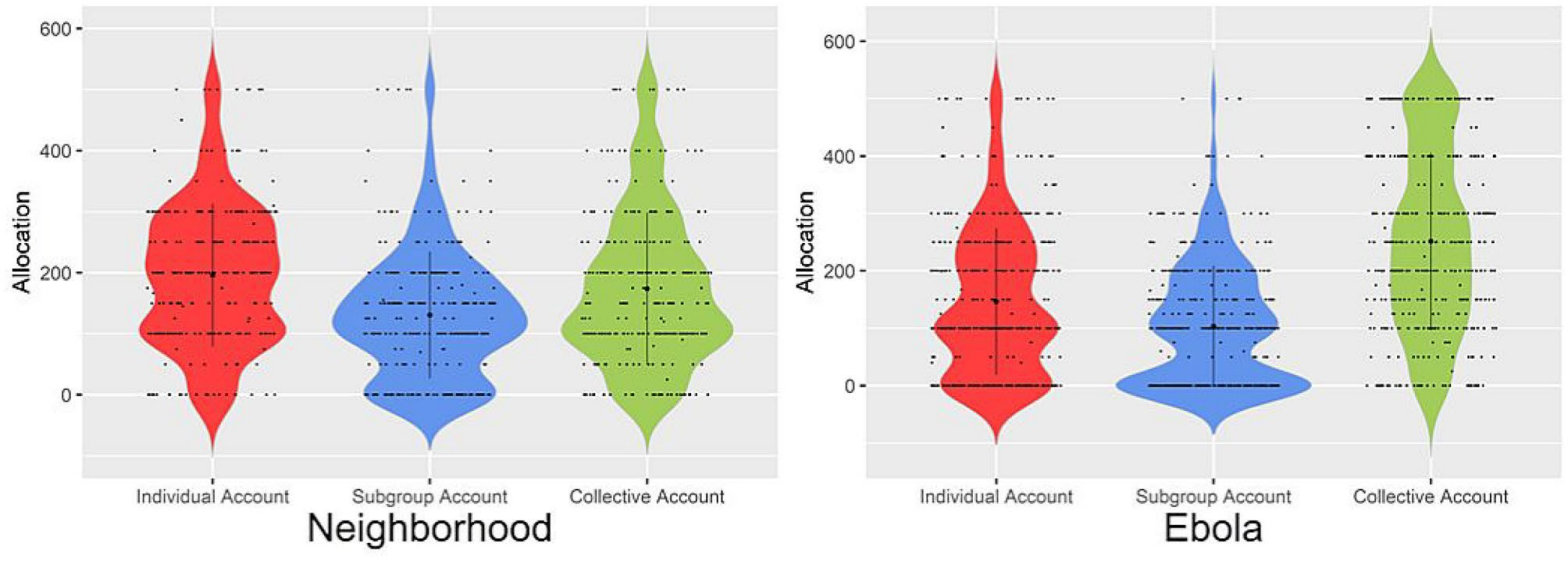
Allocations to individual, subgroup, and collective accounts in the Neighborhood and Ebola scenarios. Dots represent individual data points across all four studies.

In both contexts there were significant differences in allocations across account types (neighborhood: χ^2^_F_(2) = 31.01, *p* < 0.001; Ebola: χ^2^_F_(2) = 36.54, *p* < 0.001). In the neighborhood improvement context, allocations to the individual account were higher (*M* = 206.41, *SD* = 114.71) than those to the subgroup account (*M* = 115.74, *SD* = 85.96; *T* = 1240.50, *z* = − 6.45, *p* < 0.001) and somewhat higher than those to the collective account, although this was not significant (*M* = 179.22, *SD* = 121.70; *T* = 4227.00, *z* = − 1.61, *p* = 0.11). Allocations to the collective account were significantly higher than those to the subgroup account, *T* = 3623.50, *z* = − 4.02, *p* < 0.001. In the Ebola context, allocations to the collective account were higher (*M* = 216.94, *SD* = 137.57) than those to the individual account (*M* = 173.84, *SD* = 118.06), *T* = 7125.50, *z* = − 2.09, *p* = 0.04, and the subgroup account (*M* = 113.01, *SD* = 97.22), *T* = 6070.50, *z* = − 5.94, *p* < 0.001, replicating the findings of Studies 2 and S1. Table 3 and Fig. 2 show allocations broken down by account type and NSD context for all three studies, plus the replication Study S1. In the neighborhood improvement context, participants tended to allocate most resources to the individual account. However, in the epidemic context, with incentives that were identical to the neighborhood improvement scheme, participants made larger allocations to the collective account.

**Table 3:** Mean allocations to individual, subgroup, and collective account across different studies.

Study	Neighborhood NSD			Ebola NSD		
	Individual account	Subgroup account	Collective account	Individual account	Subgroup account	Collective account
Study 1	184.59_a_	147.72_b_	167.69_ab_	–	–	–
Study 2	–	–	–	127.10_a_	97.68_a_	275.99_b_
Study S1	–	–	–	149.93_a_	97.07_b_	254.00_c_
Study 3	206.41_a_	115.74_b_	179.22_a_	173.84_a_	113.01_a_	216.94_b_

Cell means within a row not sharing a common subscript differ significantly (*p* < 0.05) in Wilcoxon Signed Ranks tests. Post hoc tests within rows were preceded by Friedman tests of the effect of account types.

Expectations of outgroup allocations to the collective account and ingroup norms about how much one should contribute to the collective account were significantly higher in the Ebola context than in the neighborhood context (see Table 4 and Supplemental Materials for full results).

**Table 4:** Means (M), standard deviations (SD) and correlations between participants’ own allocations to collective account, their expectations of others’ allocations to this account, and prescriptive norms in Study 3 (numbers above the diagonal represent correlations in the neighborhood context, numbers below the diagonal represent correlations in the Ebola context).

Measure	*M* (*SD*) Neighborhood	*M* (*SD*) Ebola	1	2	3	4
1. Allocations	179.21_a_ (121.70)	216.94_b_ (137.57)	–	0.33***	0.34***	0.66***
2. Expectations—outgroup	103.23_a_ (82.54)	157.74_b_ (141.24)	0.52***	–	0.66***	0.28***
3. Expectations—ingroup	110.12 (79.00)	109.31 (101.44)	0.23**	0.29***	–	0.27**
4. Norms—ingroup	199.07_a_ (148.10)	266.12_b_ (147.07)	0.59***	0.44***	0.19**	–

***p < 0.001. Means within rows not sharing a common subscript differ significantly (*p* < 0.05).

#### Correlations between allocations, expectations, and norms

Table 4 shows correlations between participants’ allocations to the collective account and their expectations and prescriptive norms about allocations to this account. Consistent with the previous findings, all correlations were significant. A multiple regression analysis showed that outgroup expectations and ingroup norms were significant predictors of allocations to the collective account (see Supplemental Materials for details).

## Discussion

We compared allocations in nested social dilemmas in two contexts, a neighborhood improvement scheme and a health care scheme during an Ebola epidemic. In the neighborhood improvement context (Study 1) we replicated Wit and Kerr^17^: Making the individual, subgroup, or collective category salient resulted in larger allocations to the corresponding account. However, this effect did not replicate when a structurally equivalent nested social dilemma was presented in an Ebola context (Studies 2 and S1). Instead, participants made larger allocations to the collective account, regardless of category salience. In Study 3 we directly compared the two contexts and again found that allocations to the collective account were higher than those to the individual or subgroup accounts in the Ebola context, but not in the neighborhood improvement context.

We also measured expectations about how ingroup and outgroup members would allocate their resources, and perceptions of how ingroup members should allocate their resources. Allocations to the collective account were consistently correlated with ingroup and outgroup expectations and with ingroup norms. Ingroup norms were also the most consistent predictor of allocations to the collective account across studies, in line with the view that the increased prosociality in the epidemic context reflects the operation of benevolence norms^9^. All the studies reported here were conducted prior to the Covid-19 pandemic, so it was not the case that responses to the Ebola context were informed by participants’ experiences during the pandemic.

The results suggest that epidemics evoke different expectations and norms about how resources should be allocated. In our studies, the Ebola context overrode the influence of other experimental manipulations. An epidemic is something that potentially affects everyone, making interdependence salient. Thinking about one’s own interests makes less sense when there is a shared fate, as protecting others enhances everyone’s chances of avoiding illness. As the United Nations High Commissioner for Refugees put it in relation to the Covid-19 pandemic, ‘no one is safe until everyone is safe’^23^. Under such circumstances, social norms prescribe greater benevolence. This is reflected in how participants in the present studies allocated resources, investing in a collective solution rather than one that helps oneself or one’s own group.

These results are also consistent with evidence that higher levels of globalization are associated with stronger tendencies to cooperate with distal others^12^, and that perceptions of common fate attenuate the association between ingroup identification and outgroup helping^24^. Epidemics or pandemics may increase such connectedness or shared fate. For example, recent evidence shows that the COVID-19 pandemic has increased support for a Universal Basic Income (UBI), a policy providing for those most in need^25^.

The current studies have certain limitations. We examined expectations of ingroup and outgroup behavior and perceptions of ingroup norms, but, apart from outgroup expectations in Study 3, these constructs were measured rather than manipulated. Thus, although they were consistently correlated with allocations, we cannot infer a causal relationship. Correlations between expectations of outgroup behavior and contributions to the collective account suggest that prosocial behavior in an epidemic context is driven partly by expectations of how others will act. However, ingroup norms were the most consistent correlate of allocations to the collective account, and these norms were higher in the Ebola context than in the neighborhood improvement context, supporting the view that an epidemic context evokes prosocial norms.

Another limitation is that participants divided a hypothetical resource. This leaves open the possibility that their decisions would be different if the circumstances and the resources were real^26,27^. This issue was investigated by Lozano^28^, who found no significant differences in cooperative behavior depending on whether the incentive was hypothetical or real, although other research suggests that participants are less generous when playing with real resources^29–31^. It is important to highlight that the study introducing the nested social dilemma involved incentivized decision-making^17^ and that Study 1 replicated these original results in a hypothetical scenario. This suggests that the hypothetical division of resources observed in the current studies would be similar if choices of participants had been incentivized. Given the consistency of the present results, it seems implausible that the difference between the neighborhood improvement and Ebola contexts would be eliminated when using real incentives. Furthermore, there are ethical concerns that constrain the experimental investigation of how an epidemic affects decision-making. Nevertheless, further research is needed to examine whether the findings observed here in two specific scenarios generalizes to other epidemic and non-epidemic contexts, other social dilemmas, and other measures of prosocial decision-making.

In conclusion, we show that an epidemic elicits more prosocial behavior compared to a neighborhood improvement context. When the interests of individuals, their subgroup, and a wider collective, are pitted against each other, an epidemic context leads to a greater preference to serve the collective. One of the key policy recommendations in a recent analysis of how social science can support adaptive responses to the Covid-19 pandemic is that “a shared sense of identity or purpose can be encouraged by addressing the public in collective terms and by urging ‘us’ to act for the common good” (p. 471)^32^. In the light of extreme epidemics being likely in the coming decades^33^, it is encouraging that such contexts should help to foster a shared sense of identity and thereby promote action for the common good.

## Methods

All studies were carried out in accordance with the guidelines of the American Psychological Association and received ethical approval from Cardiff University’s School of Psychology Research Ethics Committee and from the Institutional Review Board at the University of Wisconsin-Madison. Informed consent was obtained from all subjects.

### Study 1

#### Participants and design

One hundred sixty British participants were recruited and compensated with course credit. Screening questions explicitly assessed participants’ attention and included instructions to click a specific button or move to the next page. The final sample included 134 participants (121 female; *M*_age_ = 19.68 years, *SD* = 2.93) who correctly answered four attention checks. We aimed to recruit as many participants as possible before the end of the semester, with a minimum of 60, the sample size used by Wit and Kerr (17). The design involved two factors: account type (individual, subgroup, collective; within-subjects) and salient categorization level (individual, *N* = 45; two-subgroups, *N* = 48; one-collective, *N* = 41; between-subjects).

#### Procedure

Participants completed an online questionnaire (see https://osf.io/f6xub/?view_only=6116fdaa7d3c4509a3c3370fd0f669c3 for all stimuli). After providing demographic information, participants were asked to imagine owning a house in a neighborhood composed of two streets. Approximately half of the 100 inhabitants, along with the participants, live on ‘Butler Street,’ and the other half on ‘South Street.’ Residents own their houses and have similar incomes. Each homeowner was to receive a £500 tax discount, as part of a City Council initiative to improve the neighborhood. This money had to be spent on making the house, the street, or the neighborhood more attractive. The 100 inhabitants of the neighborhood comprised the superordinate collective, and each of the two streets formed one of the two subgroups.

The £500 could be divided among three accounts: a private account, a subgroup (street) account, and a collective (neighborhood) account. Each participant could use the £500 to repaint their own house or contribute to two accounts used to repaint houses at a discounted price. The first of these was for homeowners living on participant’s own street. Allocations to this subgroup fund would qualify for a 60% reduction in the price of repainting houses on this street. The second account was shared between homeowners living on both streets. Allocations to this collective fund would secure a 75% reduction on repainting houses in the entire neighborhood. The 60% and 75% reductions are equivalent to the coefficients that Wit and Kerr (2002) used for the subgroup and the collective account.

Participants then answered questions about the extent to which the collective account would be helpful in producing different outcomes (see Supplemental Materials). Next, participants were randomly assigned to one of three experimental conditions and asked to imagine that they had decided to participate in a competition for a cash prize and were motivated to win it. In the individual condition, the contest involved the participant competing with other homeowners in the city trying to make their houses as attractive as possible. In the two-subgroups condition, the contest rewarded the most spectacular street improvement in a neighborhood. This condition involved homeowners on one street competing with homeowners on the other street. In the one-collective condition, the contest involved the two streets trying to make the neighborhood as attractive as possible and competing with other neighborhoods.

Participants were asked to divide their £500 between the individual account, the subgroup account, and the collective account in any way they wished, provided their allocations summed to £500. This was followed by questions asking about the extent to which participants’ interests were at odds with the interests of other homeowners (see Supplemental Materials). Three further questions asked about the expected allocations of the homeowners living on the other street, the expected allocations of the fellow homeowners living on participants’ own street, and about how owners of properties on participants’ own street should spend their money. In each case, participants were asked to divide the £500 between the individual, subgroup, and collective accounts. The final questions asked participants about their concern about the state of their own and other people’s properties and about how much participants thought they had in common with other homeowners (see Supplemental Materials).

### Study 2

#### Participants and design

One hundred fifty-one British students completed the study (see Supplemental Materials for details). The final sample included 126 participants (108 female; *M*_age_ = 19.58 years, *SD* = 1.60) who correctly answered four attention checks. The design involved two factors: account type (individual, subgroup, collective; within-subjects) and salient level of categorization (individual, *N* = 44; two-subgroups, *N* = 42; one-collective, *N* = 40; between-subjects).

#### Procedure

In an online questionnaire, participants were asked to imagine that they were working in the British Embassy in Abidjan, Ivory Coast. Half the 100 embassy employees are Ivorian citizens, the other half are British citizens. The two groups were said to have similar pay. The 100 embassy employees composed the superordinate collective, while the British and Ivorian employees comprised the two subgroups.

The instructions explained that special measures were being taken by the UK government to cope with the epidemic: each embassy employee would receive £500 to cover additional health expenses. This money had to be spent on health and could be divided among three accounts: a private account, a subgroup (British employees) account, and a collective (all embassy employees) account. By contributing to the subgroup or collective accounts, Ebola treatments could be purchased at a discount. Allocations to the subgroup account would earn a 60% reduction in the price of Ebola treatments (participants were told that there was a similar, separate fund for the Ivorian employees). Allocations to the collective fund would secure a 75% discount on Ebola treatments. By contributing to the subgroup and collective accounts, employees had less control over the money but also gained an opportunity to maximize their investment, provided others also contributed. Participants were randomly assigned to one of three experimental conditions. In the individual condition, they were told that they would have to compete with other embassy employees for access to health care. In the two-subgroups condition, participants were told that the British embassy employees would have to compete with the Ivorian employees for access to health care. In the one-collective condition, all embassy employees would have to compete with other institutions for access to health care.

Participants were then asked to divide their £500 between their individual account, the subgroup account, and the collective account in any way they wished, provided the allocations summed to £500. They then provided the expected allocations of Ivorian employees, the expected allocations of fellow British employees, and indicated how British employees should spend their money. Each of these questions asked participants to divide the £500 between the individual, subgroup, and collective accounts. Participants also answered questions about the extent to which different accounts would be helpful in obtaining specific outcomes, about the extent to which participants’ interests were in competition with the interests of other people, about participants’ concern about their own and others’ welfare, and about how much participants thought they had in common with others (see Supplemental Materials).

### Study 3

#### Participants and design

American students (*N* = 417) completed the study. We aimed to recruit 50 participants per condition. The final sample included 334 participants who correctly answered three attention checks (232 female; *M*_age_ = 18.83 years, *SD* = 0.80, group sizes ranging from 34 to 49). The study had a 2 × 4 design with context (neighborhood vs. Ebola) and information about outgroup contribution to the collective account (no information; low allocation; low allocation and positive emotion; low allocation and negative emotion) as between-subjects variables.

#### Procedure

Participants completed the study online. The two contexts were identical to the ones used in the previous studies (with the exception that the material referred to US dollars rather than British pounds, and the embassy in the Ebola context was a US embassy rather than a British embassy). Participants were randomly assigned to one of eight experimental conditions. In the outgroup contribution conditions participants were asked to imagine seeing a text message sent by an outgroup member (a neighbor living on the other street, or an Ivorian co-worker). This message was presented in the form of a photograph and included information about the outgroup member’s contribution and/or feelings about the contribution. In the low allocation condition, the message stated: ‘*I didn’t give much to the Neighborhood [UN] fund’*. In the positive and negative emotion conditions, messages stated: ‘*I didn’t give much to the Neighborhood [UN] fund but I feel good about that’* versus ‘*I didn’t give much to the Neighborhood [UN] fund and I don’t feel good about that’*. In the no information condition, participants were not shown any message.

Participants were then asked to divide their $500 between the individual account, the subgroup account, and the collective account such that their allocations summed to $500. They then reported expected allocations of the outgroup and ingroup members, and how the ingroup members should divide their money. Participants in the outgroup contribution conditions then rated the generosity of the outgroup member’s contribution and how the outgroup member felt about this contribution. Additional questions asked participants about their concern about their own and others’ properties or welfare and about how much they had in common with the ingroup and the outgroup (see Supplemental Materials).

## Supplementary Information

Supplementary Information.

## Data Availability

Experimental stimuli and datasets from the studies are available at: https://osf.io/f6xub/?view_only=6116fdaa7d3c4509a3c3370fd0f669c3.
