# Supplementary material for: An epidemic context elicits more prosocial decision-making in an intergroup social dilemma
Source: Sci Rep. 2022 Nov 21;12:18974. doi: 10.1038/s41598-022-22187-z (PMC9678879; doi:10.1038/s41598-022-22187-z)
Supplement: Supplementary file 1 — Supplementary Information. [file 41598_2022_22187_MOESM1_ESM.docx]

**Supplemental Materials**

**Contents**

**S1. Study S1: Replication of Study 2**

**S2. Additional Information and Measures**

Study 1

Study 2

Study S1 (Replication of Study 2)

Study 3

**References**

**Overview**

This document starts with a concise report of Study S1, which was an exact replication of Study 2, focusing on the same subset of measures that are presented in the main article. This is followed (in S2) by a description of additional measures for all studies.

**S1. Study S1: Decision-Making in the Context of an Epidemic (Replication)**

The category salience effect observed in Study 1 was not found in Study 2. Although highlighting the subgroup categorization level increased participants’ allocations to the subgroup account, allocations to the individual and collective account were not affected by the category salience manipulation. However, there was a marked tendency for allocations to differ as a function of account type: Participants made larger allocations to the collective account than to the other two accounts, a pattern that was not evident in Study 1, where allocations to the collective account (*M* = 167.69, *SD* = 127.78) did not differ markedly from allocations to the individual (*M* = 184.59, *SD* = 118.26) and subgroup accounts (*M* = 147.72, *SD* = 118.82), *T* = 2984.00, *z* = -1.13, *p* = .26 and *T* = 2977.00, *z* = -1.17, *p* = .27, respectively. To assess the robustness of the findings of Study 2, we conducted an exact replication, again manipulating category salience in the context of the Ebola epidemic.

**Method**

One hundred and fifty British participants (75 women, *M*_age_ = 48.87 years, *SD* = 12.70) were recruited via Pureprofile and completed the study online in late March-early April 2015, during the Ebola outbreak in West Africa. As in Study 2, the design involved two factors: account type (individual, two-subgroups, one-collective; within-subjects) and salient level of categorization (individual, *N* = 46; two-subgroups, *N* = 54; one-collective, *N* = 50; between-subjects). The materials and procedure were identical to those of Study 2, with the exception that all participants completed the study online.

**Results**

***Allocations***

The effect of the category salience manipulation was not significant for allocations to any of the three accounts (individual account, *F*(2, 146) = 0.33, *p* = .72, η_p_^2^ < .01; subgroup account, *F*(2, 147) = 0.83, *p* = .44, η_p_^2^ = .01; collective account, *F*(2, 147) = 1.26, *p* = .29, η_p_^2^ = .02. As in Study 2, allocations varied as a function of account type, regardless of condition, χ^2^_F_(2) = 66.51, *p* < .001. Allocations to the collective account were significantly higher (*M* = 254.00, *SD* = 189.00) than those to the individual (*M* = 149.93, *SD* = 162.93), *T* = 5711.00, *z* = -3.81, *p* < .001, and subgroup accounts (*M* = 97.07, *SD* = 132.29), *T* = 4938.00, *z* = -5.95, *p* < .001. The difference between the individual and subgroup accounts was also significant, *T* = 1295.00, *z* = -3.03, *p* < .001.

***Correlations Between Allocations, Expectations, and Norms***

Table 3 displays correlations between participants’ allocations to the collective account, and their expectations and prescriptive norms about allocations to this account. Consistent with the findings of Studies 1 and 2 in the main paper, allocations to the collective account were positively correlated with expectations of outgroup and ingroup allocations to the same account, and with perceptions of how much should be allocated. Effects of category salience condition on the expectations and norms measures are described below, in S2.

Table 3

*Correlations between participants’ own allocations to collective account, their expectations of others’ allocations to this account, and prescriptive norms (Study S1*)*.*

|  | 1 | 2 | 3 | 4 |
| --- | --- | --- | --- | --- |
| 1. Allocations | - |  |  |  |
| 2. Expectations – outgroup | .48^***^ | - |  |  |
| 3. Expectations – ingroup | .66^***^ | .58^***^ | - |  |
| 4. Norms – ingroup | .76^***^ | .53^***^ | .59^***^ | - |

Note. ^***^ – *p* < .001.

**S2. Additional Measures and Analyses**

**Study 1**

**Method**

***Additional Measures***

**Comprehension Check.** After reading instructions for the NSD task, participants answered six questions about the extent to which the collective account would be helpful in producing different outcomes (obtaining maximum discounts on repainting all houses in the neighborhood, obtaining good discounts on repainting houses on participants’ own street, having full control over the money, increasing the value of all the properties in the neighborhood, increasing the value of all the properties on participant’s street, and increasing the value of participant’s own house). Responses were recorded on 7-point response scales ranging from *not at all helpful* to *very helpful*.

**Expected Allocations and Ingroup Norms.** Participants reported the expected allocations of homeowners living on other street (outgroup expectations), the expected allocations of homeowners living on their own street (ingroup expectations), and how homeowners on participants’ own street should spend their money, as a measure of ingroup norms, by dividing £500 between the different account types.

**Concern for the State of the Properties.** Four questions measured participants’ concern about the state of their own property, properties on their own street, properties on the other street, and properties in the neighborhood, on 7-point scales ranging from 1, *very little* *concern,* to 7, *a great deal of concern.*

**Perceived Similarity*.*** Three questions investigated how much participants thought they had in common with others living on the same street, on the other street, and in other parts of the city with 7-point scales ranging from *very little* to *a great deal/very much*.

**Perceived Conflict of Interest.**  Perceived conflict of interest was measured with four questions with 7-point response scales ranging from *not at all* to *very much*. Participants indicated extent to which their own interests were at odds with the interests of other homeowners on the same street (perceived conflict with ingroup), homeowners on the other street (perceived conflict with outgroup), homeowners living in the same neighborhood (perceived conflict with collective), and homeowners in other parts of the city (perceived conflict with other citizens).

***Procedure***

Participants completed the study online. After providing demographic information, participants received instructions about the NSD task, followed by the instruction checks. After dividing the £500, participants answered the questions about perceived conflicts of interest, reported their expectations about allocations of ingroup and outgroup, and indicated how they thought ingroup members should spend their money as a measure of ingroup norms. Finally, participants indicated their concern for the state of the different properties and answered questions about perceived similarity, before they were debriefed, thanked and received a compensation for their time.^[[1]](#footnote-1)^

**Results**

**Comprehension Check*.*** Participants in all conditions rated the collective neighborhood account as generally unhelpful in having full control over their £500 (*M* = 1.79, *SD* = 1.15). This rating was significantly lower than 4, the scale midpoint, *t*(133) = -22.22, *p* < .001. The collective account was rated as helpful in obtaining maximum discounts on repainting houses in all neighborhood (*M* = 6.64, *SD* = 0.66), in increasing the value of all properties in the neighborhood (*M* = 6.06, *SD* = 0.97), in obtaining good discounts on repainting houses on participant’s own street (*M* = 5.84, *SD* = 1.08), in increasing the value of houses on participant’s street (*M* = 5.62, *SD* = 1.07), and in increasing the value of participant’s own house (*M* = 5.21, *SD* = 1.28). For these items, ratings were significantly higher than 4, *t*s > 10, *p*s < .001.

***Expected Outgroup Allocations***

**Effects of Category Salience.** The category salience manipulation significantly affected participants’ expectations of how much homeowners living on the other street would contribute to the individual account, *F*(2, 131) = 14.88, *p* < .001, η_p_^2^ = .18. In the individual condition (*M* = 262.44, *SD* = 110.85), participants thought that outgroup members would keep significantly more for themselves than in the one-collective (*M* = 161.59, *SD* = 103.39) and two-subgroups (*M* = 149.48, *SD* = 109.46) conditions, both *p*s < .001. This and subsequent pairwise comparisons used the Tukey HSD correction. The difference between the two-subgroups and one-collective conditions was not significant, *p* = .86.

Expectations of how much homeowners living on the other street would allocate to subgroup account also varied as a function of condition, *F*(2, 131) = 27.65, *p* < .001, η_p_^2^ = .30. Participants in the two-subgroups condition (*M* = 267.71, *SD* = 117.82) expected these allocations to be significantly higher than the individual (*M* = 126.22, *SD* = 82.37) and one-collective (*M* = 162.80, *SD* = 77.48) conditions, both *p*s < .001. The difference between the individual and one-collective conditions was not significant, *p* = .18.

Finally, expectations of how much homeowners living on the other street would allocate to the collective account were also affected by condition, *F*(2, 131) = 10.87, *p* < .001, η_p_^2^ = .14. Participants in the one-collective condition expected the outgroup to allocate more (*M* = 175.61, *SD* = 116.12) than did those in the individual (*M* = 111.33, *SD* = 86.37) and two-subgroups (*M* = 82.81, *SD* = 82.05) conditions, *p* = .006 and *p* < .001, respectively. The difference between the individual and two-subgroups conditions was not significant, *p* = .32.

**Effects of Account Type.** Expectations of outgroup allocations significantly differed across account types, χ^2^_F_(2) = 33.18, *p* < .001. Participants expected the outgroup to favor the individual account (*M* = 191.12, *SD* = 118.87) and the subgroup account (*M* = 188.10, *SD* = 112.85), over the collective account (*M* = 120.78, *SD* = 101.88), *T* = 1884.00, *z* = -3.96, *p* < .001 and *T* = 1603.00, *z* = -4.02, *p* < .001, respectively. The difference between expected allocations to the individual and subgroup accounts was not significant, *T* = 2904.50, *z* = -0.12, *p* = .91.

***Expected Ingroup Allocations***

**Effects of Category Salience.** Participants’ expectations of how much homeowners living on their own street would contribute to the individual account varied significantly as a function of condition, *F*(2, 131) = 13.36, *p* < .001, η_p_^2^ = .17. Expectations were higher among participants in the individual condition (*M* = 248.56, *SD* = 106.75) than in the two-subgroups (*M* = 160.31, *SD* = 106.53) and one-collective (*M* = 145.12, *SD* = 89.31) conditions, both *p*s < .001. The difference between the one-collective and two-subgroups conditions was not significant, *p* = 0.76.

Expectations of how much homeowners living on participants’ street would contribute to the subgroup account also varied as a function of condition, *F*(2, 131) = 17.28, *p* < .001, η_p_^2^ = .21. Participants in the two-subgroups condition expected these allocations to be higher (*M* = 253.13, *SD* = 119.13) than did those in the one-collective (*M* = 164.63, *SD* = 88.91) individual (*M* = 140.67, *SD* = 76.21) conditions, both *p*s < .001. The difference between the individual and one-collective conditions was not significant, *p* = .49.

Finally, expectations of how much homeowners living on their own street would contribute to the collective account were also influenced by condition, *F*(2, 131) = 12.60, *p* < .001, η_p_^2^ = .16. Expectations were significantly higher in the one-collective condition (*M* = 190.24, *SD* = 127.09) than in the individual (*M* = 110.78, *SD* = 83.45) and two-subgroups (*M* = 86.56, *SD* = 88.65) conditions, *p* = .001 and *p* < .001, respectively. The difference between the individual and two-subgroups conditions was not significant, *p* = .48.

**Effects of Account Type.** Expectations of ingroup allocations significantly differed across account types, χ^2^_F_(2) = 30.40, *p* < .001. Participants expected the ingroup to favor the individual (*M* = 185.30, *SD* = 110.71) and subgroup accounts (*M* = 188.28, *SD* = 108.49) over the collective account (*M* = 126.42, *SD* = 108.86), *T* = 2067.50, *z* = -3.55, *p* < .001 and *T* = 1555.50, *z* = -3.94, *p* < .001, respectively. The difference between the individual and subgroup accounts was not significant, *T* = 2824.00, *z* = -0.31, *p* = .76.

***Norms for Ingroup Allocations***

**Effects of Category Salience.** Participants’ perceptions of how much the homeowners on their own street should contribute to the individual account were influenced by condition, *F*(2, 130) = 10.13, *p* < .001, η_p_^2^ = .13. The amount was significantly higher for participants in the individual condition (*M* = 177.73, *SD* = 118.48) than for those in the two-subgroups (*M* = 97.92, *SD* = 93.94) and one-collective (*M* = 91.59, *SD* = 83.68) conditions, *p* = .001 and *p* < .001, respectively. The difference between the two-subgroups and one-collective conditions was not significant, *p* = .95.

Perceptions of how much people living on participants’ street should contribute to the subgroup account also varied as a function of condition, *F*(2, 131) = 16.87, *p* < .001, η_p_^2^ = .20 and were significantly higher in the two-subgroups condition (*M* = 253.65, *SD* = 143.29) than in the one-collective (*M* = 134.02, *SD* = 105.01) and individual (*M* = 120.11, *SD* = 110.40) conditions, both *p*s < .001. The difference between the individual and one-collective conditions was not significant, *p* = .85.

Norms about how much homeowners from the participant’s street should contribute to the collective account were significantly influenced by the condition, *F*(2, 131) = 8.83, *p* < .001, η_p_^2^ = .12. Participants in the one-collective condition thought that their ingroup members should contribute more to the collective account (*M* = 274.39, *SD* = 141.03) than did those in the two-subgroups (*M* = 148.44, *SD* = 139.38) condition, *p* < .001. The norm for ingroup allocations was also higher in the one-collective condition than in the individual condition (*M* = 206.11, *SD* = 142.54), but the pairwise comparison did not reach conventional levels of significance, *p* = .07. The difference between the individual and two-subgroups conditions was not significant, *p* = .12.

**Effects of Account Type.** Norms for ingroup allocations significantly differed across account types, χ^2^_F_(2) = 10.43, *p* = .005. Participants thought that ingroup members should allocate more to the collective account (*M* = 206.34, *SD* = 149.02) and to the subgroup account (*M* = 172.20, *SD* = 135.52) than to the individual account (*M* = 122.37, *SD* = 106.63), *T* = 4261.50, *z* = -3.84, *p* < .001 and *T* = 3630.00, *z* = -2.72, *p* = .01, respectively. The difference between the collective and subgroup accounts was not significant, *T* = 3189.50, *z* = -1.31, *p* = .19.

***Multiple Regression Analysis***

A multiple regression analysis examined participants’ allocations to the collective account as a function of expectations about outgroup and ingroup allocations and norms about ingroup allocations. The model significantly predicted participants’ allocations, *F*(3, 130) = 73.00, *p* < .001, *R^2^_adj_* = .63. All variables were significant predictors (expectations, outgroup: *B* = 0.20, *F*(1, 130) = 3.90, *p* = .05; expectations, ingroup: *B* = 0.39, *F*(1, 130) = 15.16, *p* < .001; ingroup norms: *B* = 0.37, *F*(1, 130) = 45.04, *p* < .001). Tolerance values were larger than .10 and variance inflation factors (VIF) did not exceed 10.

**Concern for the State of the Properties*.*** Ratings of participants’ concern about the state of their own property were not affected by the category salience manipulation, *F*(2, 131) = 0.37, *p* = .69, η_p_^2^ < .01. The same was true for participants’ concern about the state of other properties on their own street, *F*(2, 130) = 0.75, *p* = .48, η_p_^2^ = .01. The effect of category salience on concern about the state of properties on the other street (*M* = 4.62, *SD* = 1.16) was marginally significant, *F*(2, 130) = 3.07, *p* = .05, η_p_^2^ = .04, such that ratings of concern tended to be higher in the one-collective condition (*M* = 5.00, *SD* = 0.96) than in the individual (*M* = 4.47, *SD* = 1.10) and the subgroup (*M* = 4.46, *SD* = 1.32) conditions, *p* = .09 and *p* = .07, respectively. The difference between the individual and two-subgroups conditions was not significant, *p* > .99. Finally, the effect of category salience on participants’ concern about the state of all properties in the neighborhood (*M* = 5.49, *SD* = 0.91) was not significant, *F*(2, 131) = 0.69, *p* = .50, η_p_^2^ = .01.

**Perceived Similarity.** Participants’ felt that they had more in common with others living on the same street (the ingroup) (*M* = 5.51, *SD* = 0.99) than with home owners living on the other street (the outgroup) (*M* = 4.79, *SD* = 0.87), *t*(132) = 8.64, *p* <.001. Ratings of how much participants felt they had in common with other people living on the same street (the ingroup) did not differ significantly as a function of category salience, *F*(2, 131) = 1.53, *p* = .22, η_p_^2^ = .02. Ratings of how much participants felt they had in common with people living on the other street (the outgroup) did vary significantly as a function of condition, *F*(2, 130) = 5.71, *p* = .004, η_p_^2^ = .08. Ratings were higher in the one-collective (*M* = 5.20, *SD* = 0.87) than in the two-subgroups (*M* = 4.77, *SD* = 1.16) and individual (*M* = 4.44, *SD* = 1.01) conditions, with a significant difference between the individual and one-collective conditions, *p* = .003. Other post-hoc tests were not significant, *p*s > .13. Finally, ratings of how much participants felt they had in common with people living in other parts of the city did not vary significantly as a function of condition, *F*(2, 131) = 0.79, *p* = .46, η_p_^2^ = .01.

**Perceived Conflict of Interest.** Participants’ perceived conflict of interest with the ingroup was not significantly influenced by category salience, *F*(2, 131) = 1.51, *p* = .22, η_p_^2^ = .02.

Participants’ perceived conflict of interest with the outgroup was significantly affected by category salience, *F*(2, 131) = 4.64, *p* = .01, η_p_^2^ = .07. Perceived conflict was highest in the two-subgroups condition (*M* = 4.87, *SD* = 1.94), followed by the individual (*M* = 4.37, *SD* = 1.60) and one-collective (*M* = 3.71, *SD* = 1.85) conditions. Follow-up pairwise comparisons revealed a significant difference between the two-subgroups and one-collective conditions, *p* = .01. The two other comparisons were not significant, *p*s > .20.

The effect of condition on perceived conflict of interest with the collective was marginally significant, *F*(2, 131) = 2.92, *p* = .06, η_p_^2^ = .04. These ratings were lowest in the one-collective condition (*M* = 3.63, *SD* = 2.12), followed by the two-subgroups (*M* = 4.38, *SD* = 1.42) and individual conditions (*M* = 4.42, *SD* = 1.50). Pairwise comparisons revealed trending differences between the individual and one-collective conditions, and between the subgroup and one-collective conditions, *p* = .08 and *p* = .10, respectively. The difference between the individual and two-subgroups conditions was not significant, *p* = .99.

Finally, there was a significant effect of condition on perceived conflict of interest with other citizens, *F*(2, 131) = 5.23, *p* = .007, η_p_^2^ = .07. Participants’ perceptions of their interests being in conflict with the interests of people living in other parts of the city were highest in the one-collective condition (*M* = 4.68, *SD* = 2.32), followed by the individual (*M* = 3.73, *SD* = 2.09) and two-subgroups (*M* = 3.21, *SD* = 2.08) conditions. A follow-up pairwise comparison between the two-subgroups and collective conditions was significant, *p* = .005. The other two comparisons did not reach conventional levels of significance, *p*s > .10.

**Study 2**

**Method**

***Additional Measures***

**Comprehension Check.** After reading the instructions of the NSD task, participants answered five questions about the extent to which the collective account would be helpful in producing different outcomes (obtaining discounts on treatments for all embassy employees, having full control over the money, obtaining discounts on treatments for British employees, protecting all embassy employees from Ebola, buying prevention kits that are not treatments). Responses were made using 7-point response scales ranging from *not at all helpful* to *very helpful.*

**Expected Allocations and Prescriptive Norms.** Participants reported the expected allocations of Ivorian employees (outgroup expectations), the expected allocations of fellow British employees (ingroup expectations), and how British employees should spend their money (ingroup norms), by dividing £500 between the different account types.^[[2]](#footnote-2)^

**Concern for Welfare.** Four questions measured participants’ concern about their personal welfare, the welfare of British embassy employees, Ivorian embassy employees, and all embassy employees, on 7-point scales ranging from 1, *very little* *concern,* to 7, *a great deal of concern*.

**Perceived Similarity*.*** Three questions investigated how much participants thought they had in common with British employees, Ivorian employees, and people living in Abidjan but not working at the embassy, with 7-point scales ranging from *very little* to *a great deal/very much*.

**Perceived Conflict of Interest.**  Participants answered four questions about the extent to which participants’ interests were at odds with the interests of British embassy employees (perceived conflict with ingroup), Ivorian Embassy employees (perceived conflict with outgroup), all other embassy employees (perceived conflict with collective), and people not working at the embassy on 7-point response scales ranging from *not at all* to *very much.*

**Miscellaneous Measures and Demographics.** Participants were asked to describe experiences of situations in which their own interests were in competition with other people’s goals and about how emotions could affect decision-making in such situations. These measures were included to inform the design of subsequent studies and will not be discussed further. Participants also completed the ‘slider’ measure of Social Value Orientation (Murphy et al., 2011) and the Mach-IV scale (Christie & Geis, 1970). These measures were included for exploratory purposes and will not be discussed further. Finally, participants provided information about their ethnicity, country of origin, and mother tongue.

***Participants and Procedure***

One hundred fifty-one British students completed the study in the laboratory (*n* = 108) or online (*n* = 43).^[[3]](#footnote-3)^ The study was conducted between October and December 2014 during the outbreak of Ebola virus disease in West Africa. Participants received instructions about the NSD task, followed by instruction checks. After dividing the £500, participants answered questions about perceived conflicts of interest, reported their expectations about allocations of ingroup and outgroup, and indicated how they thought ingroup members should spend their money as a measure of ingroup norms. Participants then indicated their concern for their personal welfare, and the welfare of their colleagues at the embassy and answered questions about perceived similarity with their different colleagues. Then the miscellaneous and demographic measures described above were administered. Finally, participants were debriefed, thanked and received compensation.

**Results**

**Comprehension Check.** Participants in all conditions rated the collective fund for all embassy employees as helpful in obtaining discounts on treatments for all embassy employees (*M* = 6.33, *SD* = 1.15). This rating was significantly higher than 4, the scale midpoint, *t*(125) = 22.67, *p* < .001. The collective fund was also rated as helpful in obtaining discounts on treatments for British employees (*M* = 5.00, *SD* = 1.44) and in protecting all embassy employees from Ebola (*M* = 5.79, *SD* = 1.11), *t*s > 7.70, *p*s < .001. The collective fund was rated as unhelpful for having full control over one’s money (*M* = 2.13, *SD* = 1.50), *t*(125) = -19.97, *p* < .001. For the question about the extent to which this fund was helpful in buying prevention kits (rather than treatments), participants’ responses were not significantly different from the scale midpoint (*M* = 3.93, *SD* = 1.81), *t*(125) = -0.44, *p* = .66.

***Expected Outgroup Allocations***

The category salience manipulation did not significantly affect participants’ expectations of how much the Ivorian employees would keep for themselves, *F*(2, 123) = 0.55, *p* = .58, η_p_^2^ < .01, how much they would allocate to their own fund, *F*(2, 123) = 1.55, *p* = .22, η_p_^2^ = .02, or how much they would allocate to the collective account, *F*(2, 123) = 0.20, *p* = .82, η_p_^2^ < .01. Expected outgroup allocations also did not significantly differ across account types, χ^2^_F_(2) = 3.15, *p* = .21.

***Expected Ingroup Allocations***

Participants’ expectations of how much the British employees would keep for themselves, allocate to the British fund, or to the collective account did not significantly vary depending on condition, *F*(2, 123) = 0.63, *p* = .53, η_p_^2^ = .01, *F*(2, 123) = 0.21, *p* = .81, η_p_^2^ < .01, and *F*(2, 123) = 0.51, *p* = .60, η_p_^2^ < .01, respectively. Expected ingroup allocations did vary across account types, χ^2^_F_(2) = 20.76, *p* < .001 and were significantly smaller for the collective account (*M* = 129.79, *SD* = 129.99) than for the subgroup (*M* = 201.55, *SD* = 123.70) and individual accounts (*M* = 168.67, *SD* = 125.90), *T* = 1603.00, *z* = -3.78, *p* < .001 and *T* = 2046.00, *z* = -2.08, *p* = .04, respectively. The difference between the individual and subgroup accounts did not reach conventional significance levels, *T* = 3132.50, *z* = -1.89, *p* = .06.

***Norms for Ingroup Allocations***

Participants’ perceptions of how much British employees should keep for themselves were not influenced by condition, *F*(2, 81) = 1.78, *p* = .17, η_p_^2^ = .04. Perceptions of how much British employees should contribute to the British fund varied as a function of condition, *F*(2, 81) = 7.59, *p* = .001, η_p_^2^ = .16, and were significantly higher in the two-subgroups condition (*M* = 150.86, *SD* = 134.71) than in the individual (*M* = 70.00, *SD* = 103.82) and one-collective (*M* = 41.35, *SD* = 78.40) conditions, *p* = .02 and *p* = .001, respectively. Norms about how British employees should contribute to the collective fund also tended to be influenced by the condition, *F*(2, 81) = 3.14, *p* = .049, η_p_^2^ = .07 and were higher for the individual condition (*M* = 389.67, *SD* = 148.43) than for the subgroup condition (*M* = 287.07, *SD* = 173.28), *p* = .05. The difference between the individual and the collective condition (*M* = 368.27, *SD* = 169.34) was not significant, *p* = .88, and the same was true for the difference between the subgroup and one-collective conditions, *p* = .16.

Norms for ingroup allocations varied across the three account types, χ^2^_F_(2) = 57.24, *p* < .001 and were significantly higher for the collective account (*M* = 347.62, *SD* = 168.02) than for the individual (*M* = 63.33, *SD* = 99.00) and subgroup accounts (*M* = 89.05, *SD* = 117.40), *T* = 2959.00, *z* = -6.85, *p* < .001 and *T* = 2357.50, *z* = -6.33, *p* < .001, respectively. The difference between the individual and subgroups accounts did not exceed conventional significance levels, *T* = 484.00, *z* = -2.01, *p* = .05.

***Multiple Regression Analysis***

A multiple regression model examining participants’ allocations to the collective account as a function of expectations about outgroup and ingroup allocations and norms about ingroup allocations was significant, *F*(3, 80) = 39.00, *p* < .001, *R^2^_adj_* = .58. Expectations about ingroup allocations and ingroup norms were significant predictors, *B* = 0.29, *F*(1, 80) = 9.16, *p* < .01 and *B* = 0.60, *F*(1, 80) = 67.47, *p* < .001, but expectations about outgroup allocations were not, *B* = 0.05, *F*(1, 80) = 0.40, *p* = .53. Tolerance values were larger than .10 and variance inflation factors (VIF) did not exceed 10.

**Concern for Welfare.** Ratings of participants’ concern about their personal welfare were influenced by the category salience manipulation, *F*(2, 123) = 3.22, *p* = .04, η_p_^2^ = .05, such that the ratings of concern were higher in the one-collective (*M* = 6.45, *SD* = 0.85) than in the two-subgroups condition (*M* = 5.86, *SD* = 1.22). There was no significant difference between the two-subgroups and individual conditions (*M* = 6.02, *SD* = 1.15), *p* = .76, and between the one-collective and individual conditions, *p* = .18.

Ratings of participants’ concern for the welfare of British employees (*M* = 5.58, *SD* = 1.20) were significantly affected by the category salience manipulation, *F*(2, 123) = 11.56, *p* < .001, η_p_^2^ = .16. Ratings of concern were higher in the one-collective condition (*M* = 6.28, *SD* = 1.20) than in the individual condition (*M* = 5.20, *SD* = 1.36), *p* < .001. Ratings of concern in the two-subgroups condition (*M* = 5.58, *SD* = 1.20) did not significantly differ from those in the two other conditions, *p*s > .66.

The effect of condition on participants’ concern for the welfare of Ivorian employees (*M* = 5.61, *SD* = 1.16) was also significant, *F*(2, 122) = 9.36, *p* < .001, η_p_^2^ = .13, such that ratings in the one-collective condition (*M* = 6.23, *SD* = 0.84) were higher than ratings in the individual (*M* = 5.39, *SD* = 1.20) and two-subgroups conditions (*M* = 5.26, *SD* = 1.17), both *p*s < .01. The difference between the two-subgroups and individual conditions was not significant, *p* = .86.

Finally, participants’ ratings of concern for the welfare of all embassy employees (*M* = 5.87, *SD* = 1.05) were also significantly affected by condition, *F*(2, 122) = 4.71, *p* = .01, η_p_^2^ = .07. Again, ratings were higher in the one-collective condition (*M* = 6.28, *SD* = 0.79) than in the individual (*M* = 5.64, *SD* = 1.22), *p* = .01, and two-subgroups conditions (*M* = 5.74, *SD* = 0.96), *p* = .05. The difference between the individual and two-subgroups conditions was not significant, *p* = .89.

**Perceived Similarity.** Participants’ perceptions of how much they had in common with other British employees (the ingroup) were significantly higher (*M* = 5.10, *SD* = 1.40) than their perceptions of how much they had in common with Ivorian employees (the outgroup) (*M* = 4.33, *SD* = 1.45), *t*(125) = 6.42, *p* < .001. These ratings did not vary significantly as a function of category salience, *F*(2, 123) = 0.92, *p* = .40, η_p_^2^ = .01 and *F*(2, 123) = 1.00, *p* = .37, η_p_^2^ = .02, respectively. Participants’ ratings of how much they had in common with people not working at the embassy also did not vary as a function of category salience, *F*(2, 123) = 0.62, *p* = .54, η_p_^2^ = .01.

**Perceived Conflict of Interest.** Participants’ perceptions of how much their interests were at odds with the interests of the ingroup (British employees), *F*(2, 123) = 0.60, *p* = .55, η_p_^2^ = .01, the outgroup (Ivorian employees), *F*(2, 123) = 0.10, *p* = .90, η_p_^2^ < .01, and the collective (all embassy employees), *F*(2, 123) = 0.19, *p* = .83, η_p_^2^ < .01, were not affected by category salience.

Participants’ perceptions of their interests being at odds with people not working at the embassy were highest in the one-collective condition (*M* = 3.70, *SD* = 2.05), followed by the two-subgroups (*M* = 3.26, *SD* = 1.89) and individual (*M* = 2.51, *SD* = 1.80) conditions. The effect of category salience was statistically significant, *F*(2, 122) = 3.75, *p* = .03, η_p_^2^ = .06, with a significant pairwise comparison between the individual and one-collective conditions, *p* = .02. The two other comparisons were not significant, *p*s > .20.

**Study S1**

**Method**

Measures in Study S1 were similar to those in Study 2, with some minor exceptions. After reading the instructions of the NSD task, participants answered six (rather than five) questions asking about the extent to which the collective account would be helpful in producing different outcomes (obtaining maximum discounts on treatments for all embassy employees, obtaining good discounts on treatments for British employees, having full control over the money, protecting all embassy employees from Ebola, protecting the British employees from Ebola, and protecting oneself from Ebola). As in Study 2, responses were made using 7-point response scales ranging from *not at all helpful* to *very helpful.* There was also a wording change in one of the ratings of competing interests: Instead of rating the extent to which their interests were at odds with the interests of people not working in the embassy, participants reported that for employees of local, Ivorian institutions.^[[4]](#footnote-4)^

**Results**

**Comprehension Check.** Participants in all conditions rated the collective fund for all embassy employees as helpful in obtaining maximum discounts on treatments for all embassy employees (*M* = 6.16, *SD* = 1.26). This rating was significantly higher than 4, the scale midpoint, *t*(149) = 20.92, *p* < .001. The collective fund was also rated as helpful in obtaining discounts on treatments for British employees (*M* = 5.66, *SD* = 1.47), protecting all embassy employees from Ebola (*M* = 6.09, *SD* = 1.23), protecting the British employees from Ebola (*M* = 5.78, *SD* = 1.38), and protecting oneself from Ebola (*M* = 5.67, *SD* = 1.56), *t*s > 13.00, *p*s < .001. The collective fund was also rated as unhelpful for having full control over one’s money (*M* = 3.23, *SD* = 2.03), *t*(148) = -4.65, *p* < .001.

***Expected Outgroup Allocations***

The category salience manipulation did not significantly affect participants’ expectations of how much the Ivorian employees would keep for themselves, *F*(2, 146) = 0.13, *p* = .88, η_p_^2^ < .01. Similarly, expectations of how much the Ivorian employees would allocate to their own fund and to the collective account for all embassy employees did not vary as a function of category salience, *F*(2, 147) = 0.29, *p* = .75, η_p_^2^ < .01 and *F*(2, 147) = 0.01, *p* = .99, η_p_^2^ < .001, respectively.

Expected outgroup allocations did vary across accounts, χ^2^_F_(2) = 23.02, *p* < .001 and were significantly higher for the individual account (*M* = 237.92, *SD* = 189.83) than for the subgroup (*M* = 131.03, *SD* = 145.22) and collective accounts (*M* = 132.63, *SD* = 167.96), *T* = 1893.00, *z* = -4.16, *p* < .001 and *T* = 2578.00, *z* = -3.61, *p* < .001, respectively. The difference between the subgroup and collective accounts was not significant, *T* = 2025.00, *z* = -0.09, *p* = .93.

***Expected Ingroup Allocations***

Participants’ expectations of how much the British employees would keep for themselves, allocate to the British fund, or allocate to the collective account did not significantly vary as a function of category salience, *F*(2, 147) = 2.06, *p* = .13, η_p_^2^ = .03, *F*(2, 146) = 0.42, *p* = .66, η_p_^2^ < .01, and *F*(2, 147) = 1.74, *p* = .18, η_p_^2^ = .02, respectively. The effect of account type on expected ingroup allocations did not exceed conventional significance levels, χ^2^_F_(2) = 5.40, *p* = .07.

***Norms for Ingroup Allocations***

Participants’ perceptions of how much British employees should keep for themselves were not influenced by category salience, *F*(2, 147) = 0.07, *p* = .93, η_p_^2^ = .001. The same was true for perceptions of how much British employees should contribute to the British fund, *F*(2, 147) = 1.15, *p* = .32, η_p_^2^ = .01, and how British employees should contribute to the collective fund, *F*(2, 147) = 0.41, *p* = .66, η_p_^2^ = .01.

Norms for ingroup allocations varied across account types, χ^2^_F_(2) = 30.56, *p* < .001 and were significantly higher for the collective account (*M* = 266.67, *SD* = 191.88) than for the individual (*M* = 138.33, *SD* = 162.47) and subgroup accounts (*M* = 95.00, *SD* = 124.55), *T* = 6588.00, *z* = -4.45, *p* < .001 and *T* = 4617.00, *z* = -6.50, *p* < .001, respectively. The difference between the individual and subgroups accounts was also significant, *T* = 1467.50, *z* = -2.34, *p* = .02.

***Multiple Regression Analysis***

A multiple regression model examining participants’ allocations to the collective account as a function of expectations about outgroup and ingroup allocations as well as norms for ingroup allocations was significant, *F*(3, 146) = 88.83, *p* < .001, *R^2^_adj_* = .64. Expectations about ingroup allocations and ingroup norms were significant predictors, *B* = 0.36, *F*(1, 146) = 24.89, *p* < .001, and *B* = .56, *F*(1, 146) = 81.30, *p* < .001, respectively. The effect of expectations of outgroup allocations was not significant, *B* = -.02, *F*(1, 146) = 0.05, *p* = .82. Tolerance values were larger than .10 and variance inflation factors (VIF) did not exceed 10.

**Concern for Welfare.** Ratings of participants’ concern about their personal welfare were not influenced by the category salience manipulation, *F*(2, 146) = 0.02, *p* = .98, η_p_^2^ < .001. The same was true for participants’ concern for the welfare of British employees, *F*(2, 147) = 0.24, *p* = .78, η_p_^2^ < .01, for the welfare of Ivorian employees, *F*(2, 147) = 0.77, *p* = .46, η_p_^2^ = .01, and for the welfare of all embassy employees, *F*(2, 146) = 0.31, *p* = .73, η_p_^2^ < .01.

**Perceived Similarity.** Participants’ perceptions of how much they had in common with other British employees (*M* = 5.30, *SD* = 1.50) were significantly higher than perceptions of how much they had in common with Ivorian employees (*M* = 4.58, *SD* = 1.63), *t*(149) = 5.96, *p* < .001. These ratings did not vary significantly as a function of category salience, *F*(2, 147) = 0.12, *p* = .88, η_p_^2^ < .01 and *F*(2, 147) = 1.59, *p* = .21, η_p_^2^ = .02, respectively. The same was true for participants’ ratings of how much they had in common with people not working at the embassy (*M* = 3.62, *SD* = 1.52), *F*(2, 146) = 0.19, *p* = .83, η_p_^2^ < .01.

**Perceived Conflict of Interest.** Participants’ perceptions of how much their interests were at odds with the interests of the ingroup (British employees), *F*(2, 147) = 1.09, *p* = .34, η_p_^2^ = .01, the outgroup (Ivorian employees), *F*(2, 146) = 1.88, *p* = .16, η_p_^2^ = .02, and the collective (all embassy employees) , *F*(2, 147) = 1.33, *p* = .27, η_p_^2^ = .02, were not affected by category salience. The same was true for perceptions of participants’ interests being at odds with the interests of employees of local, Ivorian institutions, *F*(2, 147) = 0.31, *p* = .73, η_p_^2^ < .01.

**Study 3**

**Method**

***Additional Measures***

Measures in Study 3 were similar to those of Study 1 (neighborhood context) and Study S1 (Ebola context), with only minor changes. Following the instructions of the NSD task, participants rated the extent to which the collective account would be helpful in producing different outcomes. After dividing their $500 between the different account types, participants in the outgroup contribution conditions rated the outgroup member’s contribution to the collective account and rated both the generosity of this contribution, using a 7-point response scale ranging from *not at all generous* to *very generous*, and how the outgroup members felt about the contribution, using a 7-point response scale ranging from *not at all happy* to *very happy*. Participants then reported the expected allocations of outgroup members, the expected allocations of ingroup members, and how ingroup members should allocate their money. The seven questions that followed asked participants about their concern about the state of their own and others’ properties [or their own and others’ welfare, depending on context], and about how much they had in common with the ingroup and the outgroup.^[[5]](#footnote-5)^

**Results**

***Ratings of Generosity and Happiness***

In the three conditions in which participants received information about the low contribution made by the outgroup member, they rated this contribution as significantly lower than 4, the scale midpoint, in terms of generosity, *M* = 2.41, *SD* = 1.39, *t*(255) = -18.13, *p* < .001. Moreover, participants rated the outgroup member as significantly less happy in the negative emotion condition (*M* = 2.38, *SD* = 1.42) than in the positive emotion condition (*M* = 5.97, *SD* = 1.08), *t*(206) = 20.47, *p* < .001.

***Comprehension Check***

**Neighborhood Context.** The collective account was rated as helpful in obtaining maximum discounts for repainting houses in all of the neighborhood (*M* = 6.30, *SD* = 1.10), in increasing the value of all properties in the neighborhood (*M* = 5.35, *SD* = 1.57), in obtaining good discounts on repainting houses on participant’s own street (*M* = 6.17, *SD* = 0.95), in increasing the value of houses on participant’s street (*M* = 5.80, *SD* = 1.31), and in increasing the value of participant’s own house (*M* = 5.60, *SD* = 1.12). For these items, ratings were significantly higher than 4, *t*s > 42, *p*s < .001. Participants rated the collective neighborhood account as generally unhelpful in having full control over their $500 (*M* = 2.31, *SD* = 1.78). This rating was significantly lower than 4, the scale midpoint, *t*(152) = 16.02, *p* < .001.

**Ebola Context.** Participants in all conditions rated the collective fund for all embassy employees as generally helpful in obtaining maximum discounts for treatments for all embassy employees (*M* = 6.30, *SD* = 1.21). This rating was significantly higher than 4, the scale midpoint, *t*(180) = 69.98, *p* < .001. The collective fund was also rated as helpful in obtaining discounts on treatments for American embassy employees (*M* = 5.50, *SD* = 1.37), protecting all embassy employees from Ebola (*M* = 5.84, *SD* = 1.33), protecting the American employees from Ebola (*M* = 5.43, *SD* = 1.36), and protecting oneself from Ebola (*M* = 5.06, *SD* = 1.66), *t*s > 41.00, *p*s < .001. The collective fund was also rated as unhelpful for having full control over one’s money (*M* = 2.28, *SD* = 1.68), *t*(180) = 18.19, *p* < .001.

***Expected Outgroup Allocations***

**Effects of Context and Information Condition.** Participants’ expectations of how much the outgroup members would allocate to the collective account were analyzed using a 2 (context: neighborhood, Ebola) x 4 (information about outgroup contribution: no information; low allocation to collective account; low allocation and positive emotion; low allocation and negative emotion) between-subjects ANOVA. Expectations were higher in the Ebola context (*M* = 157.74, *SD* = 141.24) than in the neighborhood context (*M* = 103.23, *SD* = 82.54), *F*(1, 324) = 17.82, *p* < .001, η_p_^2^ = .05. The main effect of outgroup contribution was also significant, *F*(3, 324) = 3.73, *p* = .01, η_p_^2^ = .03. The mean expectation in the no information condition was 170.93 (*SD* = 141.84); in the low allocation condition it was 107.13 (*SD* = 110.24); in the low allocation and positive emotion condition it was 124.63 (*SD* = 117.86); and in low allocation and negative emotion condition it was 133.43 (*SD* = 108.10). The difference between the low allocation and no information conditions was significant, *p* = .003, and there was also a tendency for expectations to be lower in the positive emotion condition than in the no information condition, *p* = .06. There were no other significant differences between conditions. The interaction between context and information about outgroup contribution was not significant, *F*(3, 324) = 0.85, *p* = .47, η_p_^2^ < .01.

**Effects of Account Type.** In both contexts, there were significant differences in expectations across account types (neighborhood: χ^2^_F_(2) = 85.57, *p* < .001; Ebola: χ^2^_F_(2) = 11.20, *p* = .004). In the neighborhood context, participants expected the outgroup to favor the individual account (*M* = 241.38, *SD* = 114.14) over the subgroup account (*M* = 158.59, *SD* = 101.94; *T* = 1634.50, *z* = -5.15, *p* < .001) and the collective account (*M* = 103.23, *SD* = 82.54; *T* = 1069.00, *z* = -7.88, *p* < .001). Expected allocations to the collective account were lower than expected allocations to the subgroup account, *T* = 1315.00, *z* = -4.72, *p* < .001.

In the Ebola context, although expected allocations to the individual account (*M* = 178.08, *SD* = 120.17) tended to be higher than expected allocations to the subgroup (*M* = 165.09, *SD* = 122.97) and the collective accounts (*M* = 157.74, *SD* = 141.24), none of the pairwise comparisons reached conventional levels of significance, all *p*s > .11.

***Expected Ingroup Allocations***

**Effects of Context and Information Condition.** A 2 (context: neighborhood, Ebola) x 4 (information about outgroup contribution: no information; low allocation to collective account; low allocation and positive emotion; low allocation and negative emotion) between-subjects ANOVA revealed no significant effects of context, *F*(1, 324) = 0.01, *p* = .91, η_p_^2^ < .001, information condition, *F*(3, 324) = 1.11, *p* = .34, η_p_^2^ = .01, or their interaction, *F*(3, 324) = 0.52, *p* = .67, η_p_^2^ < .01.

**Effects of Account Type.** In both contexts, there were significant differences in expectations across account types (neighborhood: χ^2^_F_(2) = 68.08, *p* < .001; Ebola: χ^2^_F_(2) = 75.62, *p* < .001). In the neighborhood context, participants expected the ingroup to favor the individual account (*M* = 220.23, *SD* = 107.31) over the subgroup account (*M* = 169.65, *SD* = 93.46; *T* = 2022.00, *z* = -3.56, *p* < .001) and the collective account (*M* = 110.12, *SD* = 79.00; *T* = 1440.00, *z* = -6.87, *p* < .001). Expected allocations to the collective account were lower than expected allocations to the subgroup account, *T* = 1407.00, *z* = -5.04, *p* < .001. In the Ebola context, expected ingroup allocations to the individual account (*M* = 223.86, *SD* = 115.27) were higher than those to both the subgroup account (*M* = 166.83, *SD* = 115.77), *T* = 3895.50, *z* = -3.96, *p* < .001, and the collective account (*M* = 109.31, *SD* = 101.45), *T* = 1793.50, *z* = -7.28, *p* < .001. Participants also expected the ingroup to allocate more to the subgroup account than to the collective account, *T* = 2701.00, *z* = -4.28, *p* < .001.

***Norms for Ingroup Allocations***

**Effects of Context and Information Condition.** A 2 (context: neighborhood, Ebola) x 4 (information about outgroup contribution: no information; low allocation to collective account; low allocation and positive emotion; low allocation and negative emotion) between-subjects ANOVA revealed a significant main effect of context, *F*(1, 324) = 16.55, *p* < .001, η_p_^2^ = .05. Specifically, participants thought that the ingroup should allocate more in the Ebola (*M* = 266.12, *SD* = 147.07) than in the neighborhood context (*M* = 199.07, *SD* = 148.10). Neither the main effect of the information condition nor the interaction effect were significant, *F*(3, 324) = 0.31, *p* = .81, η_p_^2^ < .01 and *F*(3, 324) = 0.49, *p* = .69, η_p_^2^ < .01, respectively.

**Effects of Account Type.** Norms did not significantly vary across account types in the neighborhood context, χ^2^_F_(2) = 1.94, *p* = .38, but there was a significant association with account type in the Ebola context, χ^2^_F_(2) = 62.74, *p* < .001. Specifically, normative allocations to the collective account (*M* = 266.12, *SD* = 147.07) tended to be higher than those to the individual account (*M* = 126.19, *SD* = 112.16, *T* = 10668.00, *z* = -6.82, *p* < .001) and to the subgroup account (*M* = 107.68, *SD* = 98.72, *T* = 8136.00, *z* = -7.81, *p* < .001). The difference between the individual and subgroup accounts was not significant, *T* = 3453.50, *z* = -1.61, *p* = .11.

***Multiple Regression Analysis***

**Neighborhood Context.** A multiple regression model examining participants’ allocations to the collective account as a function of expectations about outgroup and ingroup allocations as well as norms for ingroup allocations was significant, *F*(3, 147) = 43.96, *p* < .001, *R^2^_adj_* = .46. Ingroup norms were a significant predictor, *B* = 0.50, *F*(1, 147) = 94.08, *p* < .001, but the effects of expected ingroup and outgroup allocations did not reach significance, *B* = 0.18, *F*(1, 147) = 2.03, *p* = .16 and *B* = 0.12, *F*(1, 147) = 1.10, *p* = .30.

**Ebola Context.** A multiple regression model examining participants’ allocations to the collective account as a function of expectations about outgroup and ingroup allocations, and norms for ingroup allocations was significant, *F*(3, 177) = 45.19, *p* < .001, *R^2^_adj_* = .42. Expectations about outgroup allocations and ingroup norms were significant predictors, *B* = 0.30, *F*(1, 177) = 23.05, *p* < .001, and *B* = 0.41, *F*(1, 177) = 47.40, *p* < .001, respectively. The effect of expectations of ingroup allocations was not significant, *B* = .08, *F*(1, 177) = 1.06, *p* = .30. Tolerance values were larger than .10 and variance inflation factors (VIF) did not exceed 10.

**Concern for State of the Properties and Welfare.** Ratings of participants’ concern were analyzed using a 2 (context: neighborhood, Ebola) x 4 (information about outgroup contribution: no information; low allocation to collective account; low allocation and positive emotion; low allocation and negative emotion) between-subjects ANOVA.

Ratings of participants’ concern about their personal interest tended to be higher in the neighborhood context (*M* = 5.83, *SD* = 1.32) than in the Ebola context (*M* = 5.56, *SD* = 1.48), *F*(1, 324) = 3.01, *p* = .08, η_p_^2^ = .01. Neither the main effect of the information condition nor the interaction were significant, *F*(3, 324) = 0.60, *p* = .61, η_p_^2^ = .01 and *F*(3, 324) = 1.41, *p* = .01, η_p_^2^ = .01, respectively.

Ratings of participants concern about their ingroup’s interests were not influenced by context, *F*(1, 321) = 0.03, *p* = .86, η_p_^2^ < .001, information condition, *F*(3, 321) = 2.03, *p* = .11, η_p_^2^ = .02, or their interaction, *F*(3, 321) = 0.65, *p* = .58, η_p_^2^ < .01.

Concern about the outgroup’s interests was higher in the Ebola context (*M* = 5.36, *SD* = 1.30) than in the neighborhood context (*M* = 4.61, *SD* = 1.30), *F*(1, 323) = 26.57, *p* < .001, η_p_^2^ = .08. The main effect of the information condition and the interaction were not significant, *F*(3, 323) = 0.67, *p* = .57, η_p_^2^ = .01 and *F*(3, 323) = 1.06, *p* = .36, η_p_^2^ = .01, respectively.

Finally, feelings of concern about the interests of the entire collective were not affected by the context, *F*(1, 323) = 1.28, *p* = .26, η_p_^2^ < .01, the information condition, *F*(3, 323) = 0.73, *p* = .53, η_p_^2^ < .01, and the interaction, *F*(3, 323) = 0.91, *p* = .44, η_p_^2^ < .01.

**Perceived Similarity.** Participants’ perceptions of how much they had in common with other members of their ingroup (neighborhood context: *M* = 5.34, *SD* = 1.28, Ebola context: *M* = 5.08, *SD* = 1.41) were significantly higher than participants’ perceptions of how much they had in common with members of the outgroup (neighborhood context: *M* = 4.72, *SD* = 1.43, *t*(150) = 6.38, *p* < .001, Ebola context: *M* = 3.98, *SD* = 1.63, *t*(180) = 9.36, *p* < .001).

Perceptions of having things in common with ingroup members tended to be higher in the neighborhood context (*M* = 5.34, *SD* = 1.28) than in the Ebola context (*M* = 5.08, *SD* = 1.41), *F*(1, 324) = 3.19, *p* = .07, η_p_^2^ = .01. Neither the main effect of the information condition, nor the interaction were significant, *F*(3, 324) = 0.65, *p* = .58, η_p_^2^ < .01 and *F*(3, 324) = 0.77, *p* = .51, η_p_^2^ < .01, respectively.

Feelings of having things in common with the outgroup were also higher in the neighborhood context (*M* = 4.72, *SD* = 1.43) than in the Ebola context (*M* = 3.98, *SD* = 1.63), *F*(1, 324) = 19.35, *p* < .001, η_p_^2^ = .06. The main effect of the information condition and the interaction effect did not reach conventional levels of significance, *F*(3, 324) = 0.33, *p* = .81, η_p_^2^ < .01 and *F*(3, 324) = 2.55, *p* = .06, η_p_^2^ = .02, respectively.

Finally, ratings of having things in common with people not living in the neighborhood or not working at the embassy were again higher in the neighborhood context (*M* = 4.26, *SD* = 1.20) than in the Ebola context (*M* = 3.06, *SD* = 1.48), *F*(1, 324) = 66.21, *p* < .001, η_p_^2^ = .17. The main effect of the information condition was also significant, *F*(3, 324) = 3.31, *p* = .03, η_p_^2^ = .03, such that participants’ ratings were highest in the low allocation and negative emotion condition (*M* = 4.00, *SD* = 1.64), followed by the no information condition (*M* = 3.54, *SD* = 1.41), the low allocation condition (*M* = 3.45, *SD* = 1.33), and the low allocation and positive emotion condition (*M* = 3.43, *SD* = 1.49). Post-hoc pairwise comparisons revealed significant differences between the positive and negative emotion condition, *p* = .03 and between the negative emotion condition and the low allocation condition, *p* = .03. Other comparisons were not significant, *p*s > .13. Finally, the interaction of context and information condition did not reach conventional significance levels, *F*(3, 324) = 2.11, *p* = .10, η_p_^2^ = .02.

**References**

Aquino, K., & Reed, A. (2002). The self-importance of moral identity. *Journal of Personality and Social Psychology*, *83*(6), 1423-1440. doi: 10.1037/0022-3514.83.6.1423

Christie, R., & Geis, F. (1970). *Studies in Machiavellianism*. New York, NY: Academic Press.

Murphy, R. O., Ackermann, K. A., & Handgraaf, M. (2011). Measuring social value orientation. *Judgment and Decision Making*, *6*(8), 771-781.

1. Participants also completed a measure of Social Value Orientation (Murphy et al., 2011), the Mach-IV scale (Christie & Geis, 1970), and the measure of Self-Importance of Moral Identity (Aquino & Reed, 2002). These measures were included for exploratory purposes and will not be discussed further. [↑](#footnote-ref-1)
2. As the result of an oversight, ratings of how British employees should spend their money were only available for 84 participants. [↑](#footnote-ref-2)
3. Study administration mode did not interact with category salience condition for participants’ allocations to the collective account, expectations for ingroup and outgroup allocations to this account, and for ingroup norms for allocations to this account, all *F*s < 2, all ps > .15. [↑](#footnote-ref-3)
4. In addition to the measure of Social Value Orientation (Murphy et al., 2011) and the Mach-IV scale (Christie & Geis, 1970), participants also completed the measure of Self-Importance of Moral Identity (Aquino & Reed, 2002). As in previous studies, these measures were included for exploratory purposes and will not be discussed further. [↑](#footnote-ref-4)
5. As in previous studies, participants completed the ‘slider’ measure of Social Value Orientation (Murphy et al., 2011) and the Mach-IV scale (Christie & Geis, 1970). These measures were included for exploratory purposes and will not be discussed further. [↑](#footnote-ref-5)
